# In vitro antimicrobial activity of Sodium hypochlorite, Chlorhexidine gluconate and Octenidine Dihydrochloride in elimination of microor- ganisms within dentinal tubules of primary and permanent teeth

**DOI:** 10.4317/medoral.17566

**Published:** 2011-12-06

**Authors:** Resmiye E. Tirali, Haluk Bodur, Gülden Ece

**Affiliations:** 1 DDS, PhD. Assistant Professor, Department of Pediatric Dentistry, Faculty of Dentistry, University of Baskent, Ankara, Turkey; 2 DDS. Assistant Professor Associate Professor, Department of Pediatric Dentistry, Faculty of Dentistry, University of Gazi, Ankara, Turkey; 3 DDS, PhD. Consultant, Department of Microbiology, School of Medicine, University of Gazi, Ankara, Turkey

## Abstract

The aim of this study was to evaluate the effectiveness of different irrigation solutions at different time intervals for the elimination of E.faecalis and C.albicans penetrated into the dentine tubules of primary and permanent teeth in vitro. The 4 mm primary and permanent teeth sections were sterilized and contaminated with a mixture of E.faecalis and C.albicans strains. After the application of different irrigation solutions (Sodium hypochlorite, Chlorhexidine gluconate, Octenidine Dihydrochloride, saline) to the contaminated tooth sections according to study groups, neutralizers were applied for inactivation of the solutions after 30 sec, 1 min and 5 min. Dentine shavings were placed into TSB and 10µL from each tube was inoculated on agar plates, followed by an incubation period of 24h at 37°C. The colonies were counted macroscopically. The results were compared by using Kruskal-Wallis and Mann Whitney U tests, with a significance level at p<0.05. Among the irrigation solutions that were tested against E.faecalis on primary and permanent teeth, the most effective one was found as 5-minute application of 0.1% Octenidine Dihydrochloride. The antibacterial effects of the tested solutions on the same time periods against C.albicans revealed no significant difference. There were no statistically significant differences between primary and permanent teeth with respect to the antimicrobial activity of the tested solutions. Moreover, Octenidine Dihydrochloride may be used as an alternative endodontic irrigant.

** Key words:**Chlorhexidine gluconate, dentine tubules, irrigation solutions, Octenidine Dihydrochloride, Sodium hypochlorite.

## Introduction

Routine endodontic procedures such as instrumentation of the pulp space may remove a limited part of the infecting bacteria and their substrate of necrotic pulp debris. In addition to this situation with the anatomical complexities of many root canals and the limitations in access by therapeutic agents to the microcanal system, this can be quite challenging (1).

Furthermore, microorganisms may remain in the dentinal tubules, grooves and other irregularities of the root canal system. Once they remain in sufficient numbers, and under an adequately supportive environment, they may multiply and reestablish clinical contamination of the pulp space (2). Therefore, several irrigation solutions have been recommended for use in combination with root canal preparation. An irrigant serves to flush out debris from the instrumented root canals, dissolve organic tissue remnants, disinfect the root canal space, provide lubrication during instrumentation and remove smear layer without causing irritation to the biological tissues (3).

Premature loss of a primary tooth may cause many functional problems (4). One of the treatment choices of pulp ally-involved primary teeth is non-vital pulp therapy. There are a few data exist regarding the use of irrigation solutions for the endodontic therapy of primary teeth (5).

The most commonly investigated agent, which has been accepted as the gold standard for the irrigation of root canal space, is sodium hypochlorite (NaOCl) because of its clinical efficacy in endodontic therapy (3). It has solvent activity for both necrotic and vital tissues (6). However there are still some concerns with respect to the toxic effects (7), bad smell and taste, corrosive potential (8) and allergic reactions (9).

Chlorhexidine gluconate, has been suggested as an endodontic irrigant because of its antibacterial effects, and lower cytotoxicity but greater substantivity than NaOCl, and efficient clinical performance (10).

Octenidine hydrochloride (OCT), developed by Sterlig Winthrop Research Institute, is a bispyridine derivative, i.e., N,N’-[1,10-decanediyldi-1(4H)-pyridinyl-4-ylidene] bis(1-octanamine) dihydrochloride. The existing data suggest that a mouthrinse containing 0.1% OCT may be capable of exerting beneficial clinical effects upon plaque accumulation and gingivitis (11). OCT used in the form of mouth rinse was reported to inhibit dental plaque and caries both in rats (12) and humans (11). It has been demonstrated that OCT appears to be more effective than chlorhexidine as a means for prolonged bacterial anti-adhesive activity (13). OCT has been suggested as an endodontic irrigant based on its antimicrobial effects and lower cytotoxicity (14).

E.faecalis was chosen as one of the test microorganisms in this experiment for the following reasons: (I) it is a well-recognized pathogen associated with persistent apical periodontitis in endodontically treated teeth (15); (II) it is resistant to NaOCl, especially at low concentrations (16); (III) it readily colonizes in dentinal tubules and can penetrate to the entire width of dentin; and (IV) it is easy to culture and it grows rapidly (17).

The other test microorganism was C.albicans for this experiment. Microbiological investigations have shown that yeast may be present in the microflora of apical periodontitis (18) and cause persistent apical periodontitis (19). Its ability to invade dentinal tubules and resistance to commonly used intracanal medicaments may help to explain why C.albicans has been associated with the cases of persistent root canal infections (20).

The aim of this study was to evaluate the effectiveness of various irrigation solutions at different time intervals for the elimination of E.faecalis and C.albicans penetrated into the dentine tubules of primary and permanent teeth in vitro.

## Material and Metods

Eighty primary and eighty permanent extracted single rooted human teeth were used for this experiment. Their crowns and the apical parts were removed with a water-cooled diamond and the fragments standardized to a length of 4 mm. They were placed in 0.5 % NaOCl for 24hr to implement surface disinfection. The internal diameter was then standardized by enlarging the root canals with an ISO 033 round bur (Horico, Berlin, Germany). The specimens were kept in sterile saline throughout the whole experimental procedures to avoid dehydration. The smear layer formed in the canal walls during endodontic instrumentation was removed by soaking in an ultrasonic bath with 17 % EDTA (Pulpdent, Watertown, USA) for 5 min followed by the application of 5.25 % NaOCl (Gazi University, Faculty of Pharmacy, Ankara, Turkey) for 5 min. The specimens, were placed in test tubes (20 primary or permanent teeth per tubes) containing 3ml of Brain Heart Infusion Broth (BHIB, Fluka, BioChemika 53286 Buchs, Switzerland) and autoclaved three times at 121°C for 30 min.

**Figure 1:**
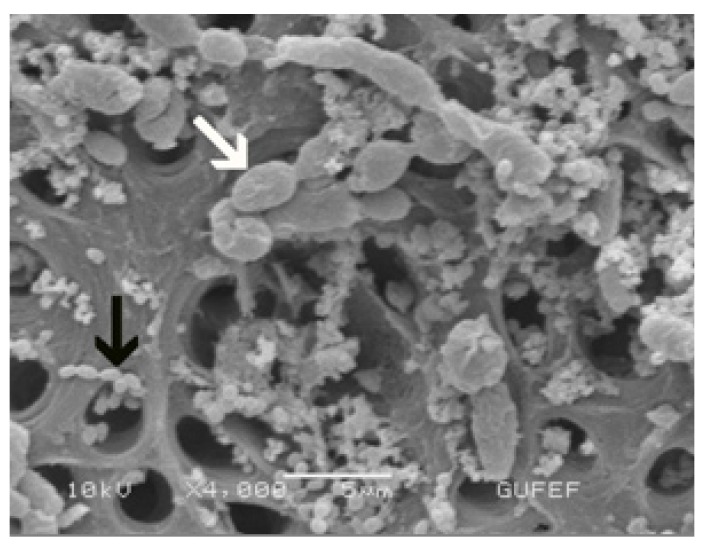
Photomicrograph of root canals contaminated with E.faecalis ( black arrow) and C.albicans (white arrow). (x4000).

C.albicans (ATCC 10231) were cultivated in Saboroud Dextrose (SDA, Biolab 20500, Budapest, Hungary) broth and E.faecalis (ATCC 29212) were cultivated in Tryptone Soya Broth (TSB, Lab M 0655052, Bury, Lancashire), respectively for 48 h and then cultured in Brain Heart Infusion Broth (BHIB, Fluka, Bio Chemika, 53286 Buchs, Switzerland) in an anaerobic chamber at 37°C for 48 h. Suspensions of C.albicans and E.faecalis had the optical density adjusted spectrophotometrically to approximately 1.5x108 colony-forming units (cfu) mL-1. Then 1.5 mL of C.albicans suspension and 1.5 mL of E.faecalis suspension were collected in a single tube containing approximately 1.5x108 mL-1 of each microorganism. Sterile pipettes were used to remove 3mL sterile BHI from test tubes and replace it with 3mL of bacterial inoculum. The tubes were closed and kept at 37°C for 21 days. The medium was changed every 3 days to ensure viability of the microorganisms. The purity of the cultures was checked at intervals. To confirm root canal infection, Four specimens (2 for primary, 2 for permanent teeth) were submitted to the same initial instrumentation and contamination procedures, and were then observed under scanning electron microscopy (SEM; JSM 6400, Tokyo, Japan; (Fig. 1)

After infection, the specimens were randomly divided into 10 groups (each group contains 8 permanent and 8 primary teeth), according to the intracanal irrigants and different time intervals.

• Group 1: 1 mL 5.25% NaOCl → 30 sec

• Group 2: 1 mL 5.25% NaOCl → 1 min

• Group 3: 1 mL 5.25% NaOCl → 5 min

• Group 4: 1 mL 0.1% Oct → 30 sec

• Group 5: 1 mL 0.1% Oct → 1 min

• Group 6: 1 mL 0.1% Oct → 5 min

• Group 7: 1 mL 2% CHX → 30 sec

• Group 8: 1 mL 2% CHX → 1min

• Group 9: 1 mL 2% CHX → 5min

• Group 10: 1 mL saline → 5min

The solutions prepared for neutralizing the tested irrigants in order to prevent continued action of the irrigants were ; the mixture of 3% Tween 80 (Merk, Darmstadt, Germany), 0.3% Lecithin (Aklar Kimya, Ankara, Turkey) and 0.1% Cystein (Merk, Darmstadt, Germany) was used for Octenisept (OCT,0.1% Octenidine hydrochloride; Schülke & Mayr, Nordersdedt, Germany) (21) while 0.6 % Sodium thiosulfate (Aklar Kimya, Ankara, Turkey) was used for NaOCl and 0.3% Lecithin and 3% Tween 80 were used for Chlorhexidine gluconate (CHX, Drogsan, Ankara, Turkey) (22) ( Table 1).

**Table 1:**
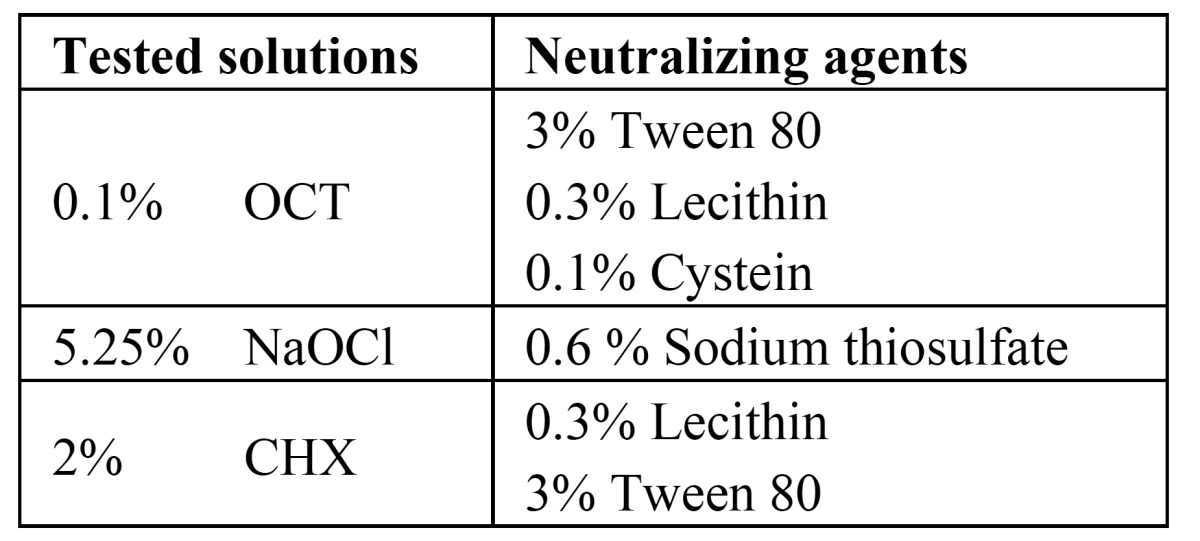
The tested irrigating solutions with the corresponding neutralizing agents.

The tooth sections were held with sterile clamps and irrigated with syringe. At the end of each test period, sterile round burs (ISO 035) were used for cutting from the irrigated surface. The specimens were held with steril clamps. The dentin shavings obtained with each bur were collected in a separate test tube containing 1 mL of TSB broth. The burs were weighed before and after the collection of dentine samples in an attempt to standardize the volume of the dentine chips removed by each bur (0.003±2x104mg). The round burs were placed in the tubes as well. After this period, 10µL of each tube was inoculated on BHIB agar plates, and left at 37ºC for 24 hours in anaerobical gaseous condition to investigate all possible bacterial growth. All assays were repeated three times. The purity of the positive cultures was macroscopically evaluated. The results were compared by using Kruskal-Wallis and Mann Whitney U tests, with a significance level at p<0.05.

## Results

The medians of CFU mL-1 of E.faecalis after the application of the tested irrigation solutions at different contact times are given in ( Table 2 and  Table 3). There were no significant differences between primary and permanent teeth in any of the tested groups (p>0.05). Application of 0.1% OCT for 5 min. was shown to be the most effective way of eliminating E.faecalis that penetrated into the dentine tubules of primary and permanent teeth in vitro (p<0.05).

**Table 2:**
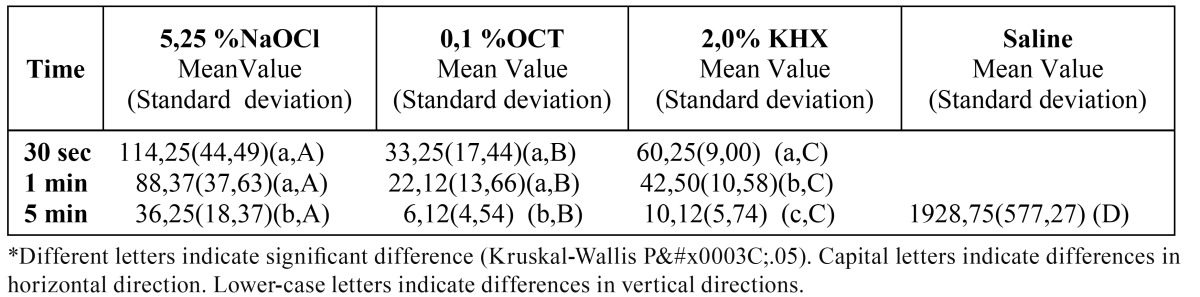
Mean values of CFU mL-1 of E.faecalis after tested irrigation solutions application to the primary teeth for different contact times.

**Table 3:**
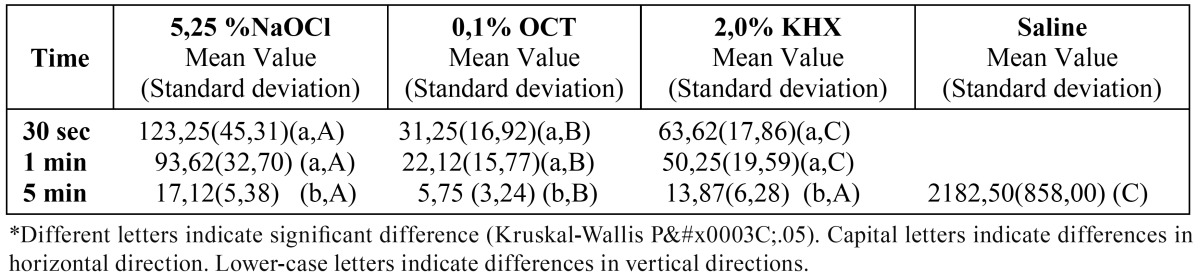
Mean values of CFU mL-1 of E.faecalis after tested irrigation solutions application to the permanent teeth for different contact times.

For primary teeth, there was a significant difference between the tested irrigation solutions. The most effective one was 0.1% OCT, while 5.25% NaOCl appeared to be the least effective irrigant. Five minute was found as the most efficient duration of application (p<0.05).

The medians of CFU mL-1 of C.albicans after the application of the tested irrigation solutions at different contact times are given in ( Table 4 and  Table 5). Similar to the results with E.faecalis, there is no significant differences between primary and permanent teeth (p>0.05). For C.albicans there isn’t significant difference between different time durations of applications. Significant differences were found between all the tested solutions and the negative control group (saline solution) (p<0.05).

**Table 4:**
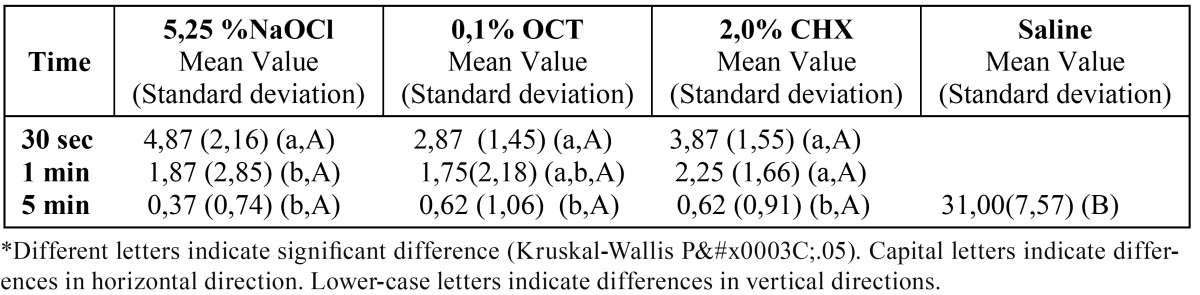
Mean values of CFU mL-1 of C.albicans after tested irrigation solutions application to the primary teeth for different contact times.

**Table 5:**
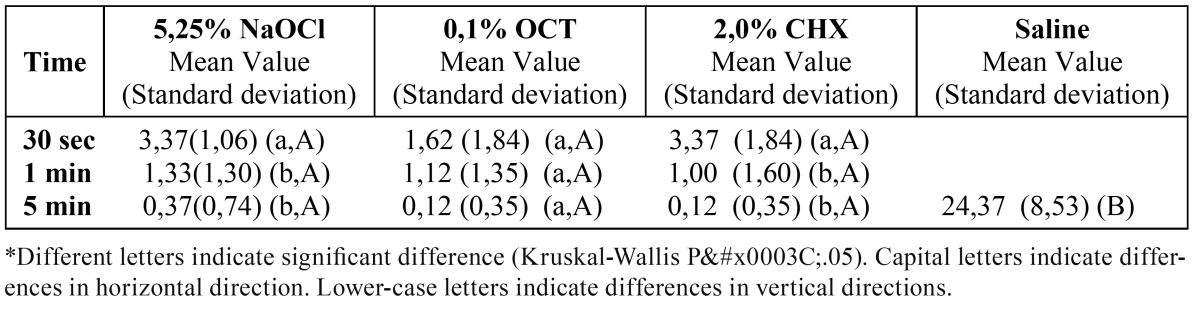
Mean values of CFU mL-1 of C.albicans after tested irrigation solutions application to the permanent teeth for different contact times.

## Discussions

The bovine dentin model was modified as using human teeth in this study for testing the efficiency of medicaments (17). Recurring periapical inflammation of root-filled teeth may occur from viable bacteria residing within the root canal system and dentinal tubules (23). With the other test methods; for example collecting microbiological samples with absorbent paper cones; the samples may only be taken from the root canal microorganisms, while it is not possible to take samples from the ones that are located inside the dentine tubules. The results of the saline treated negative control specimens found in the present study confirmed the predictability of the infection of the dentinal tubules with E.faecalis and C.albicans.

The effectiveness of irrigation solutions is directly related to the concentration as well as the volume (24). The choice of irrigant should be one that rapidly exerts its antimicrobial activity against resistant microorganisms found in the root canal and dentinal tubules. In the present study, all the tested solutions significantly reduced the microorganisms within dentinal tubules in a period of 5 min.

Several researchers have pointed out the potential advantages of CHX as an antimicrobial medicament in endodontic therapy (6,17,25-28). CHX is a broad-spectrum antimicrobial agent (29), that can be used effectively as an irrigant (6,25-27), disinfect the dentinal tubules (17,28) , and be absorbed into the dentin (27).

As mentioned before; OCT, is a mouthrinse capable of exerting beneficial clinical effects upon plaque accumulation and gingivitis development (11). OCT has been demonstrated to be more effective than chlorhexidine as a means for prolonged bacterial antiadhesive activity (13).

Although quantitative bacterial reduction after chemomechanical preparation was significant, the samples treated with NaOCl were still positive regarding the presence of cultivable bacteria. If the antimicrobial activity was assumed as the only requirement of an endodontic irrigant, we could say according to the results of this study Octenidine can be preferred as an irrigantion solution. However, sodium hypochlorite possesses another very significant attributes that Octenidine is not known to posses. It has been reported that sodium hypochlorite has the ability to dissolve pulp tissues (6) and most clinicians consider the dissolution of pulp tissue by an irrigant to be of primary importance in the root canal instrumentation. Nevertheless, Octenidine may still be useful as an alternative endodontic irrigant. Its excellent antimicrobial properties support this inference.

Although preventive measures have reduced caries incidence, the premature loss of pulpally involved primary teeth remains to be a common problem. There are many morphologic changes that continually occur within primary teeth root canal anatomy. The root canal systems of primary molars frequently contain many ramifications and deltas between the canals rendering thorough debridement quite difficult (30). Therefore, the irrigation procedure for primary teeth is as important as permanent teeth.

It is worth emphasizing, however, that further clinical studies to assess the behavior of primary teeth submitted to endodontic therapy using the suggested auxiliary chemical solutions are of fundamental importance.

Additional studies will be needed to investigate Octenidine’s relative safety and absence of unfavorable cosmetic and organoleptic properties. In addition, more information will be needed concerning probable dose-effectiveness, optimal regimens and modes of action like antibiofilm strategies. Thus results of this study may not express the actual efficacy of a medicament against microorganisms forming biofilm. These findings suggest that Octenidine may be useful as an endodontic irrigant. Its antimicrobial properties indicate it could be useful substitude in patients who are allergic to sodium hypochlorite.
